# Prospective evaluation of hydroxychloroquine in pediatric interstitial lung diseases: Study protocol for an investigator-initiated, randomized controlled, parallel-group clinical trial

**DOI:** 10.1186/s13063-020-4188-4

**Published:** 2020-04-03

**Authors:** Matthias Griese, Meike Köhler, Sabine Witt, Daniela Sebah, Matthias Kappler, Martin Wetzke, Nicolaus Schwerk, Nagehan Emiralioglu, Nural Kiper, Kai Kronfeld, Christian Ruckes, Hans Rock, Gisela Anthony, Elias Seidl

**Affiliations:** 1 Department of Pediatrics, Dr. von Hauner Children´s Hospital, University Hospital, LMU Munich, German Center for Lung Research (DZL), Lindwurmstraße 4, 80337 Munich, Germany; 2 Department of Pneumonology, Medical School Hannover, Hannover, Germany; 3 Department of Pediatric Pulmonology, Hacettepe University Faculty of Medicine, Ankara, Turkey; 4 IZKS, Interdisciplinary Center for Clinical Trials, University Medical Center Mainz, Mainz, Germany; 5 Department of Neurology, University of Marburg, Central Information Office, Marburg, Germany

**Keywords:** chILD, Interstitial lung disease, Hydroxychloroquine

## Abstract

**Background:**

Interstitial lung diseases in children (chILD) are rare and consist of many different entities that affect the parenchyma of the lungs, leading to a chronic lung disease. The natural course of many of these diseases is connected with a high morbidity and significant mortality. Symptomatic treatment consists of oxygen supplementation, adequate nutrition adapted to the high energy demand generated by the disease due to the increased breathing effort required, as well as immunization against respiratory pathogens to prevent exacerbations through respiratory infections. No proven pharmacological treatments are available to date. This placebo-controlled study aims to evaluate the efficacy and safety of the mid-term use of hydroxychloroquine in chILD.

**Methods and design:**

The study is an explorative, prospective, randomized, double-blind, placebo-controlled investigation of hydroxychloroquine (HCQ) in chILD. Patients can be included into the trial when diagnosed with a chronic (≥ 3 weeks’ duration) diffuse parenchymal lung disease (chILD) (1) genetically defined, (2) histologically defined or (3) diagnosed with idiopathic pulmonary hemorrhage (hemosiderosis). The study contains of two different study blocks, a START and a STOP block, which can be initiated in any sequence. Each patient can participate in each block only once. In the START block subjects are randomized to parallel groups for 4 weeks treatment, then the placebo group is switched to the active drug. In the STOP block, subjects taking HCQ are randomized into parallel groups treated with placebo or HCQ.

**Discussion:**

This study is the first international, investigator-initiated, prospective and controlled investigation of a pharmacological treatment in chILD. The block design was selected as it has the advantage of accommodating patients who are initiating or withdrawing from HCQ therapy, thus allowing the participation of those who were previously started on off-label HCQ. The cross-over design and selected outcome parameters enables us to include appropriate numbers of patients of all age groups from neonates to adults suffering from these rare diseases.

**Trial registration:**

This is an exploratory, Phase 2a, randomized, double-blind, placebo-controlled, parallel-group, multinational study investigating the initiation or withdrawal of hydroxychloroquine in subjects with chILD. Study title: Hydroxychloroquine in pediatric ILD: START randomized controlled in parallel groups, then switch placebo to the active drug, and STOP randomized controlled in parallel groups to evaluate the efficacy and safety of hydroxychloroquine (HCQ). Short title: HCQ in pediatric ILD, particularly 4surfdefect. EudraCT, ID: 2013–003714-40. Registered on 2 July 2013. ClinicalTrials.gov, ID: NCT02615938. Registered on 8 November 2015. IZKS trial code: 2013–006; Sponsor: University Hospital, Ludwig-Maximilians University of Munich. Responsible Party: Prof. Dr. med. Matthias Griese, University Hospital, Ludwig-Maximilians University of Munich, Germany.

## Background

Interstitial lung disease in children (chILD) is an umbrella term for more than 200 different entities that affect the parenchyma of the lungs, leading to a chronic lung disease. Previous studies have reported an incidence of 0.1–16 per 100,000 children per year and a prevalence of 1.3–3.6 per million children [1–3]. Often the children are dependent on oxygen (O_2_) for a long period of time or require mechanical ventilation [4]. The overall mortality in childhood is around 15% [4]. No successfully proven treatments are available and there only exist few pharmacological treatment options based on expert agreement [5] and anecdotal experience collected from individual treatments. Symptomatic treatment consists of O_2_ supplementation if necessary, adequate nutrition adapted to the high energy demand generated by the disease due to the increased breathing effort required, as well as immunization against respiratory pathogens to prevent exacerbations through respiratory infections [6]. Common drug treatments include glucocorticosteroids, hydroxychloroquine (HCQ), and steroid-sparing agents, such as azathioprine, cyclophosphamide, cyclosporine or methotrexate, are also tried [6]. A few cases with possible responses to macrolide antibiotics have been reported.

Due to the rarity of the diseases, only international studies can collect sufficient numbers of patients to evaluate treatment in controlled settings. We used the European-wide project on chILD, the chILD-EU project [7], to prepare and implement a randomized controlled, Phase 2a study to evaluate treatment with HCQ in chILD. With this study we aim to: (1) evaluate the efficacy of HCQ against placebo in chILD, (2) evaluate the safety of the mid-term use of HCQ, (3) be able to make a decision on the risks and benefits of the use of HCQ and (4) help in standardizing the pharmacological treatment of chILD.

HCQ is approved for the treatment of malaria, rheumatoid arthritis, juvenile idiopathic arthritis, lupus erythematosus and dermatological conditions caused or aggravated by sunlight [8]. Due to the assumed various immunomodulatory mechanisms of HCQ it has been used frequently for more than 40 years off-label for different autoimmune diseases (e.g., Sjögren’s syndrome and inflammatory osteoarthritis) in a wide range of clinical conditions and, in particular, in chronic or acute and often very severe pediatric diffuse parenchymal lung diseases.

Eighty-three cases of chILD were reported between 1984 and 2013 to have been treated with chloroquine (CQ) or HCQ alone or in combination with other drugs [9]. From these data it was not possible to predict which patients would benefit from the treatment. Clear positive clinical improvements, usually occurring within 4 weeks, have been reported in 15 of 16 published cases when HCQ was given alone, and in 37 of 53 published cases, when given in combination with glucocorticosteroids. Obviously, a publication bias towards successful cases has to be considered.

Applications even for many years appeared safe with very few reported side effects during off-label usage [9]. Again, a significant underreporting of side effects or unsuccessful trials has to be expected. The oral doses ranged from 3.5–10 mg/kg bodyweight per day [9]. In this respect it is notable that there exist two pharmaceutical forms of HCQ, HCQ base and the HCQ sulfate (correction factor 200 mg sulfate = 155 mg base) [9]. The majority of case reports and small series did not indicate which of the two pharmaceutical forms the dose is referring to. Hydroxychloroquine treatment may have several side effects including rare retinal changes and, most frequently, abdominal discomfort and pain. The retinal changes are dose-dependent and can be irreversible [10]. In one series of patients who received HCQ, abdominal pain in the first week of treatment was reported in a minority of cases [11].

The use of HCQ in a trial in chILD has several important challenges. Firstly, it is used in a broad age and weight range, from birth to adulthood, with not much pharmacological data available [9]. Secondly, disease severity varies from acute respiratory distress and failure to thrive to chronic, mild partial respiratory insufficiency. Randomization of acutely ill patients to treatments which may potentially be harmful may be very difficult to achieve and consent by the caregivers, even if the treatments might be helpful, may not be given, thus reducing the number of subjects that can be included. Nevertheless, patients in a chronic disease state have a different response pattern than those who are acutely sick. All this must be addressed by an appropriate study design. Thirdly, no randomized controlled studies have been performed in chILD. Therefore, outcome variables have not been used in studies and their responsiveness to treatments is unknown. Lastly, the extreme rarity and heterogeneity of the diseases targeted, as well as their wide geographical scattering, induces significant burden and huge logistic challenges for such a clinical trial. The goal of this Phase 2a study is to include all chILD patients who are planned to be, or are actively being, treated with HCQ. We intend to gather as much information as possible to improve the knowledge in this group of patients. Therefore, the character of this study is explorative and not confirmatory. It was designed to closely accommodate the current clinical care situation.

## Methods and design

### Study goals, duration and number of subjects planned

This study is an explorative, prospective, randomized, double-blind, placebo-controlled, investigator-initiated investigation of HCQ in pediatric ILD. We aim to: (1) evaluate the efficacy of HCQ against placebo in chILD, (2) assess the safety of the mid-term use of HCQ, (3) be able to make a decision on the risks and benefits of the use of HCQ and (4) help in standardizing the pharmacological treatment of chILD. Subject recruitment started in August 2015 and was expected to be low because the numbers of eligible patients were unknown. At the beginning of the study we planned to assess over 100 subjects for eligibility and to allocate 80 subjects to each study block. Recruitment and treatment of subjects was expected to be performed in up to 100 trial centers. The estimated study completion date is April 2025.

### The cross-over design

**Fig. 1:**
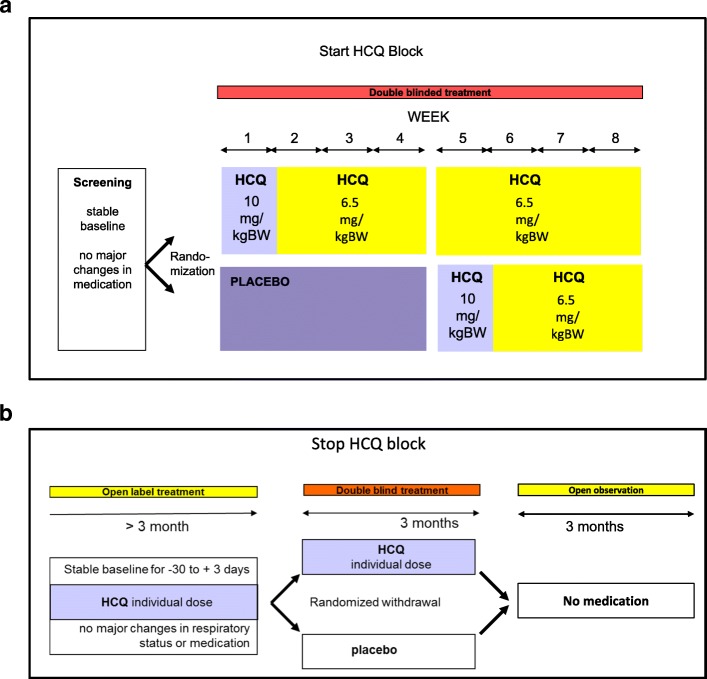
Schematic study setup. The study contains two different study blocks: a START block (**a**) and a STOP block (**b**) These blocks can be initiated in any sequence as needed by the subjects. Each patient can participate in each block only once

To include as many possibly eligible patients in the study, the study contains two different study blocks, a START block and a STOP block. These may be initiated in any sequence by the subjects. By this method, patients already taking HCQ for various time periods and with unknown drug-related benefits may also be recruited to the study. Each patient can complete each block only once. In the START block, subjects are randomized to parallel groups for 4 weeks, then the placebo group is switched to the active drug for another 4 weeks. In the STOP block, subjects taking HCQ are randomized into parallel groups treated with placebo or HCQ for 3 months to investigate the withdrawal of HCQ for assessment of its efficacy and then followed for another 3 months (Fig. 1 and Additional file 1). We expect an equal distribution between the START and STOP blocks as, optimally, each patient progresses through both study blocks.

### Schedule of the study

At the beginning and at the end of a treatment block the participants must consult an ophthalmologist to assess any disturbance of central vision, check their visual and reading acuity, and undergo a slit-lamp examination and fundoscopy. Only at the discretion of the ophthalmologist may ocular coherence tomography (OCT), fundus auto-fluorescence (FAF) imaging, stereoscopic slit-lamp examination of the retina (e.g., with a 90-D or 78-D biconvex lens), and central visual field examination using an Amsler chart (preferably red on black) or automated perimetry be performed. Evaluation may need to be extended according to signs and symptoms to include retinal photography and visual electrophysiological tests. For patients who have previously received continuous treatment for more than 5 years, an individual arrangement should be agreed with the ophthalmologist. Visual side effects are dose-related and the drug should be stopped, if they develop.

At baseline and end of the trial, as well as after 4 weeks (START block) and 12 weeks (STOP block), the participant should undergo electrocardiography testing. Any signs of conduction disorders (bundle-branch block/atrio-ventricular heart block) as well as biventricular hypertrophy can be a sign of cardiomyopathy, which has been described as occurring while receiving HCQ. In this case, further cardiological workup is necessary. If there are any signs or symptoms of cardiomyopathy, HCQ must be discontinued immediately. The echocardiography is performed to assess for pulmonary hypertension and to exclude cardiomyopathy at the beginning of the study. Any signs of hypoglycemia, like low blood glucose levels or reports of potential hypoglycemic episodes, will lead to intensive observation of blood glucose levels and, if persistent, change of medication (e.g., reduction or stopping).

### Outcome parameters

There are not yet any validated outcome parameters available for a patient’s respiratory condition in chILD. We selected as the primary outcome parameter the change in oxygenation. If no supplemental O_2_ is necessary the O_2_ saturation is measured twice over 1 min in an awake patient after 5 min at rest. The measurements have to be at least 1 min apart. The stable average value will be recorded. If the patient needs supplement O_2_, the supplementation is withdrawn after 5 min at rest and the O_2_ saturation is measured twice over 1 min. The measurements have to be at least 1 min apart. The stable average value will be recorded. If the O_2_ saturation falls below SpO_2_ 80%, O_2_ is returned to the patient and and the value of 80% is recorded. The patient’s respiratory condition can vary considerably over relatively short time periods from breathing room air to being ventilated. Therefore, oxygenation cannot be determined from O_2_ saturation alone, but we also need to consider the respiratory effort of the subject or support needed as necessary to achieve a certain O_2_ saturation. The patient is defined as a responder to the initiation or to the withdrawal of HCQ if the O_2_ saturation, respiratory rate, O_2_ airflow or the oxygenation index change to a clinically meaningful extent (Table 1). We did not include pO_2_ or pCO_2_ alone in the primary outcome to assess oxygenation because these variables very rapidly change in infants and children with crying or in different activity circumstances.

**Table 1:** Definition of responders (change of oxygenation) to treatment depending on the patient’s condition

Patient’s condition	Measured parameters	Definition of significant change or responder to the initiation of hydroxychloroquine (HCQ)	Definition of significant change or responder to the withdrawal of HCQ	Method of aggregation
In room air	SaO_2_ Respiratory rate	Increase ≥ 5% in O_2_ Sat, or Decrease in resp. rate ≥ 20%	Decrease ≥ 5% in O_2_ Sat, or Increase in resp. rate ≥ 20%	Proportion of responders
Using O_2_ supplement	SaO_2_ withdrawal Respiratory rate	Increase ≥ 5% in O_2_ Sat or Decrease in resp. rate ≥ 20% or Support no longer needed	Decrease ≥ 5% in O_2_ Sat, or Increase in resp. rate ≥ 20% or Support newly needed	Proportion of responders
Using high-flow nasal cannula	O_2_ flow, air flow	Decrease ≥ 20% or Support no longer needed	Increase ≥ 20% or Support newly needed	Proportion of responders
Ventilated	Oxygenation index	Decrease ≥ 20% or Support no longer needed	Increase ≥ 20% or Support newly needed	Proportion of responders

For secondary outcome parameters the following variables will also be investigated:

- Clinical responses: intercostal retraction, coughing, weight for height, clinical course of lung disease (since last visit), number of pulmonary exacerbations, and changes in chest x-ray as well as changes in pulmonary function tests and distance of the 6-min walking test (*if* the child is old enough to perform the tests)
- Change in co-medication: cumulative amounts of steroid equivalents
- Patient-reported outcome (self-assessment questionnaires): quality of life and health economics

All analyses of secondary outcome will be interpreted purely exploratorily.

### Safety monitoring

Safety monitoring for the drug is performed noting adverse events (AEs), clinical laboratory values (differential blood count glutamic oxaloacetic transaminase (GOT), glutamate-pyruvate transaminase (GPT), gamma glutamyl transpeptidase (gGT), creatinine, lactate dehydrogenase (LDH), potassium, creatine kinase, blood glucose levels, HCQ steady-state drug level), electrocardiography, echocardiography and ophthalmological review.

### Inclusion and exclusion criteria

Patients can be included into the trial when diagnosed with a chronic (≥ 3 weeks’ duration) diffuse parenchymal lung disease (chILD) that is: (1) genetically defined (e.g., surfactant protein B/C (SFTPC, SFTPB), ATP-binding cassette transporter 3 (ABCA3), NK2 Homeobox 1 (NKX2–1), T-box transcription factor 4 (TBX4), Niemann-Pick disease B/C (NPC2, NPC1, NPB), coatomer subunit alpha (COPA), lipopolysaccharide-responsive and beige-like anchor protein (LRBA) or others), (2) histologically defined (e.g., chronic pneumonitis of infancy, desquamative interstitial pneumonia, lipoid pneumonitis, cholesterol pneumonia, non-specific interstitial pneumonia, pulmonary alveolar proteinosis (after the exclusion of mutations in granulocyte-macrophage colony-stimulating factor-receptor (GMCSF-R)a/b and GMCSF autoantibodies), usual interstitial pneumonia, follicular bronchitis/bronchiolitis, lymphogenic interstitial pneumonia, storage disease with primary pulmonary involvement) or (3) diagnosed with idiopathic pulmonary hemorrhage (hemosiderosis). Patients have to be clinically stable with no major changes in their medication (Table 2). Exclusion criteria for patients are listed in Table 3. In an amendment, the following entities were removed to restrict the spectrum of the cohort: microvasculopathy, cryptogenic organizing pneumonia, diffuse alveolar damage/acute interstitial pneumonia, acute fibrinous and organizing pneumonia, giant-cell pneumonia, chILD clinically diagnosed (CDB-ILD), respiratory bronchiolitis-interstitial lung disease, sarcoidosis, hypersensitive pneumonitis, neuroendocrine-cell hyperplasia of infancy and pulmonary interstitial glycogenosis.

**Table 2:** Inclusion criteria for patients to participate in the study

1	Diagnosis of chronic (≥ 3 weeks duration) diffuse parenchymal lung disease chILD: a) Genetically^a^ or b) Histologically^b^ Diagnosis of chronic (≥ 3 weeks duration) idiopathic pulmonary hemorrhage (hemosiderosis)
2	If chILD genetically diagnosed: patients of all ages (including preterm babies and adults age > 30 years) If chILD histological diagnosed or diagnosis of idiopathic pulmonary hemorrhage: mature newborn (age ≥ 37 weeks of gestation age) to adults (age ≤ 30 years)
3	Patients should be clinically stable during baseline (between visits 1 and 2) for inclusion into the study^c^
4	START block: no HCQ treatment in the last 12 weeks STOP block: stable HCQ treatment for at least the last 12 weeks
5	Ability of subject or/and legal representatives to understand character and individual consequences of clinical trial
6	Signed and dated informed consent of the subject (if the subject has the ability) and the representatives (of under-age children) must be available before start of any specific trial procedures

^a^Surfactant dysfunction disorders including patients with mutations in *SFTPC*, *SFTPB*, *ABCA3*, *NKX2–1*, further extremely rare entities with specific mutations; for example, in *TBX4*, *NPC2*, *NPC1*, *NPB*, *COPA*, *LRBA* and other genes

^b^Chronic pneumonitis of infancy (CPI), desquamative interstitial pneumonia (DIP), lipoid pneumonitis/cholesterol pneumonia, non-specific interstitial pneumonia (NSIP), pulmonary alveolar proteinosis after the exclusion of mutations in granulocyte-macrophage colony-stimulating factor-receptor (GMCSF-R)a/b and GMCSF autoantibodies, usual interstitial pneumonia (UIP), follicular bronchitis/bronchiolitis/lymphogenic interstitial pneumonia (LIP), storage disease with primary pulmonary involvement (e.g., Niemann-Pick)

^c^To determine this, attending physicians can use SpO_2_ in room air for patients on room air or on O_2_ supplement; the absolute difference in SpO_2_ is expected not to be ≥ 5% between visits 1 and 2. For patients on respiratory support, the summary key parameters should not change ≥ 20% between visits 1 and 2 and no major changes in other medications between visits 1 and 2

**Table 3:** Exclusion criteria for patients to participate at the study

1.	chILD primarily related to developmental disorders; chILD primarily related to growth abnormalities reflecting deficient alveolarization^a^; chILD related to chronic aspiration; chILD related to immunodeficiency; chILD related to abnormalities in lung-vessel structure; chILD related to organ transplantation/organ rejection/GvHD; chILD related to recurrent infection
2.	Acute severe infectious exacerbations
3.	Known hypersensitivity to HCQ, or other ingredients of the capsules (lactose monohydrate, povidone, maize starch, magnesium stearate, hypromellose, macrogol or titanium dioxide (E 171), silicon dioxide or mannitol), to sucrose octaacetate or sodium saccharin
4.	Proven retinopathy or maculopathy
5.	Glucose-6-phosphate-dehydrogenase deficiency resulting in favism or hemolytic anemia, myasthenia gravis, hematopoietic disorders
	Pregnancy and lactation (women with childbearing potential have to practice a medically accepted contraception during the trial and til 3 months after the end of the treatment with HCQ, and a negative pregnancy test (serum or urine) should be existent on visit 1 if they are girls of childbearing age and only if sexual relations are known or probable. It is at the discretion of and is the responsibility of the attending physician to decide whether a pregnancy test is necessary or not. Reliable contraceptives are systematic contraceptives (oral, implant, injection). Women who are sterile through surgery can participate in the trial. At the discretion of the investigator, sexual abstinence is also accepted as a contraceptive method. Girls after the menarche must receive counseling about birth control methods in the presence of at least one parent, which has to be documented in the patient’s notes
6.	Participation in other clinical trials during the present clinical trial or not beyond the time of 4 half-lives of the medication used, at least 1 week
7.	Hereditary galactose intolerance, lactase deficiency or glucose-galactose malabsorption
8.	Renal insufficiency at screening, defined as glomerular filtration rate (GFR) < 40 mL/min/1.73 m^2^ in patients aged 3 to 8 weeks < 60 mL/min/1.73 m^2^ in patients ≥ 8 weeks of age
9.	Liver disease, gastrointestinal disorder, hematological disorder, epilepsy or other neurological disorder, psoriasis, porphyria at the discretion of the treating physician
10.	Simultaneous prescription of other potentially nephrotoxic or hepatotoxic medication at the discretion of the treating physician

List of abbreviations: *chILD*  children’s interstitial lung disease, *GvHD* graft versus host disease, *mL* milliliter, *min* minutes, *GFR* glomerular filtration rate, *HCQ* hydroxychloroquine

^a^If not diagnosed with a specific genetic cause (listed in Table 3)

### Randomization and blinding

Patients are allocated to the two treatments, orally administered HCQ and placebo, in a ratio of 1:1 by central randomization within each age group. The age-matched stratification is performed according to two age groups defined as: (1) infants aged between 3 weeks and 2 years of age and (2) children aged older than 2 years. As of an anticipated skewed frequency distribution of cases, the older ones being less frequent. The randomization procedure does not consider the sex of the patients, since no sex-specific responses to HCQ therapy have been reported yet and are not expected. The randomization list is generated by an independent institute using a validated system, which involves a pseudo-random number generator to ensure that the resulting treatment sequence will be both reproducible and non-predictable.

The study medication is packed and blinded according to the random list. Each patient medication box is sent together with the sealed emergency unblinding envelopes to the sites. At the end of the trial, any emergency opening of the envelopes will be controlled after collecting the explanations for unblinding and checking the unused treatment units. The code, which is kept confidential in the pharmacy, will be broken regularly only at the end of the study, and after checking the data, recording any protocol violations after freezing the statistics database, allowing the collected data to be analyzed.

Blinding was achieved by providing the study-specific HCQ powder/substance and placebo-powder/substance in appropriately covered capsules to keep the contents invisible for both of the study drugs, HCQ and placebo. Placebo capsules contain sucrose octaacetate to obscure the bitter taste of HCQ. In a double-blinded testing, eight healthy volunteers were not able to distinguish placebo from HCQ.

### The dose

Commonly in chILD, the HCQ sulphate is given at a dose of 10 mg/kg bodyweight per day. After extensive discussion with the lead competent authority (the German BfArM) and a European group of chILD experts, on the basis of safety data available, and extensive experience in pediatric rheumatology, the dose was set at 10 mg/kg bodyweight per day for loading during the initial week of treatment, followed by 6.5 mg/kg bodyweight per day. In the STOP block, the individual dose that a patient has been taking until enrollment should be continued. The selected duration of the placebo phase was the result of a delphi questionnaire among European pediatric pneumologists and balanced the anticipated time point, when a treatment effect could be noted in a majority of patients if present, and the time period tolerated to withhold the drug from a placebo-treated subject [9].

### The risks

The clinical research interventions and procedures planned represent an appropriate balance of risk and potential benefit. Specific measures were implemented to ensure adequate protection for minors, including parental permission and assent of able children, assurance of a direct benefit for the child and minimization of the overall risk of study participation. The risks to which the children are exposed are low, both compared to the potential therapeutic benefits and the risks of the disease. The additional burden put on the children from participation in the study is minimal. Except for a sample for HCQ blood-level measurements, no investigations or tests are performed, which would not be performed outside the study setting, including ophthalmological and cardiological investigations, as well as liver function tests.

### The statistical analysis

Due to the exploratory nature of the study there is no formal sample size calculation. The study aims to include as many patients as possible in both START and STOP blocks (optimally 80 patients in each or up to 160 participants in one block only). At least over 100 patients should be included in the study. All randomized subjects are included in the intention-to-treat (ITT) population. This population is the primary analysis population. Within the ITT population analyses, subjects will be assigned to the treatment to which they were randomized. For the combined analysis, the ITT population consists of all patients who are at least in the START or the STOP block. For analysis, subgroups of patients will be defined, based on age, categories of diagnosis and further appropriate factors. The statistical final analysis plan (SAP) will be documented before unblinding of group allocation.

### Efficacy analyses

The primary populations for the analyses of efficacy are the ITT populations (randomized patients) for each block. For the combined analysis the ITT population consists of all patients who are at least in the START or the STOP block. For all hypotheses, two-sided exploratory *p* values will be provided. If appropriate, the analyses will be adjusted for the stratification factor of the randomization (age group).

### Analysis of adverse events

All summaries and listings of safety data are performed for the safety population. Frequencies of subjects experiencing at least one adverse event (AE) are displayed by body system and preferred term according to Medical Dictionary for Regulatory Activities (MedDRA) terminology. Detailed information collected for each AE will include the description of the event, duration, whether the AE was serious, its intensity, its relationship to the trial drug, any action taken and clinical outcome. Summary tables present the number of subjects observed with AEs and corresponding percentages. Additional subcategories are based on event intensity and relationship to trial drug.

### Analysis of clinical laboratory findings

Listings are prepared for each laboratory measure and structured to permit review of the data per subject as they progress on treatment. Summary tables are prepared to examine the changes of laboratory measures over time. Additionally, shift tables are provided to examine the changes of laboratory data from normal baseline to values outside the corresponding reference range during/after treatment. HCQ levels will be assessed at the end of the study.

## Discussion

Children’s interstitial lung diseases (chILD) cover a large, heterogenic group of rare pediatric pulmonary disorders. They are difficult to diagnose, many are not yet genetically defined, there are no evidence-based pharmacological treatments and the natural disease course cannot be reliably predicted, as such information is frequently lacking. Some years ago, we implemented a web-based international European registry and biobank – the European Management Platform for Children’s Interstitial Lung Diseases (chILD EU Register) [7] to provide structures for the collection and care of such “orphaned” patients. The platform also has the technical and legal requirements to perform investigator-initiated, randomized controlled treatment trials. This study is embedded in the chILD_EU Register and is the first prospective, international, interventional study of chILD. At the beginning of the project in 2011 we did not anticipate the broad diversity and depth of the burdens and pitfalls that we were facing. Here we will discuss some decisions that we had to make from the beginning to the successful implementation of this trial.

1. Which of the chILD entities should be included into the study?

As the entities for which empiric treatment with HCQ was successful were not molecularly defined, we decided to be as inclusive as possible. Nevertheless, our inclusion criteria were clearly set to select so far: (1) subjects of all ages (premature infant to adults) with genetically defined surfactant-dysfunction syndromes or molecularly defined diseases caused by mutations in genes associated with diffuse parenchymal lung diseases and anecdotal or possible responsiveness to HCQ; (2) mature newborn (≥ 37 weeks of gestational age) to young adults with lung biopsies showing any of the histopathology pattern and clinical phenotypes known to be associated with these well-defined conditions and (3) children with idiopathic pulmonary hemosiderosis, in the absence of a lung biopsy, due to some evidence for HCQ responsiveness [12]. In an amendment, the following entities were removed: microvasculopathy, cryptogenic organizing pneumonia, diffuse alveolar damage/acute interstitial pneumonia, acute fibrinous and organizing pneumonia, giant-cell pneumonia, chILD clinically diagnosed (CDB-ILD), respiratory bronchiolitis-interstitial lung disease, sarcoidosis, hypersensitive pneumonitis, neuroendocrine-cell hyperplasia of infancy and pulmonary interstitial glycogenosis. Reasons to exclude the first six entities were that they were non-specific and non-predicting for the diagnosis of chronic diffuse parenchymal lung disease; the next three entities were excluded as there are established and specific treatment responses in adult patients and the last two have generally a favorable natural course and lack any previous data of HCQ responsiveness. These restrictions narrow the disease spectrum, thus the revised collection includes either genetically well-defined entities or complementary histopathologically defined chronic diffuse parenchymal lung diseases, previously clarified as idiopathic interstitial pneumoniae [13]. Despite some potential disadvantages associated, e.g., a great variability of disease severity as currently known, a broad range of different molecular entities, potentially new and different underlying pathomechanisms, a wide age-range from infancy into adulthood, we expected significant advantages to this “basket” approach. An important justification comes from the molecular definition of the diseases, at least allowing “*n* of 1” descriptions and later grouping of similar observations. As this Phase 2b study is exploratory, some entities with good responses may be identified and allow later focusing on these. Additionally, the approach selected here mirrors everyday care and clinical routine, i.e., treating the majority of this group of chILD patients empirically, enhancing their chances of participating in this study.

2. Can families living far away from a study center be included?

To avoid disadvantages for families and patients living in remote areas, we tried to open as many trial centers as possible. However, it was soon evident that the financial and, in particular, the administrative burden was too big and in Germany in the beginning of 2019, 12 study centers were open. While the Ethics Committee would allow expansion of a clinical trial site to another hospital (“flying doctor”) to prevent excluding patients for no good reason, the administrative office of the sponsor and the lead clinical trial unit did not agree to this approach.

3. How can we maximize participation in the trial?

As already described above, we designed separate START and STOP blocks, which was helpful to recruit subjects otherwise lost because subjects were already taking the medication. It will be interesting to analyze the results with statistical approaches, that take care of such design features. To our knowledge, similar approaches have not been used widely. Due to the heterogeneity of the diseases with a wide spectrum of clinical symptoms and severity it is not possible to determine one specific HCQ mode of duration that will fit the majority of subjects. Physicians anticipate the treatment of some patients who will be treated with HCQ only for some weeks as well as patients who will need HCQ treatment for several years. Due to the variety of clinical conditions and our intention to be as inclusive as possible in this exploratory trial, we allowed a variable time period in which patients were treated with HCQ between the START and STOP blocks. As this is an investigator-initiated trial, reimbursement of the sites for including subjects is limited and is very much dependent on the funding organizations (DZL €4000 and E-rare €6000 for START and STOP). Lastly, and despite more than 50% of those known to the study centers being recruited into the trial, it was soon evident that the number of cases observed in Germany was insufficient to complete the trial. Luckily, we could obtain an international grant, which would allow us to start to expand the study into six other European countries (Portugal, Spain, Austria, Poland, Italy and Turkey). We hope that we can successfully perform this study and that it will generate some important insights into the fascinating areas of chILD.

## Supplementary information

**Additional file 1.** a. Time schedule of the HCQ START block and trial assessments. b.Time schedule of the HCQ STOP block and trial assessments.

## Data Availability

Data generated from this study will be made available on request to the corresponding author after completing the study.

## Abbreviations

ABCA3: ATP-binding cassette transporter 3
chILD: Children’s interstitial lung disease
COPA: Coatomer subunit alpha
FAF: Fundus auto-fluorescence
GFR: Glomerular filtration rate
gGT: Gamma glutamyl transpeptidase
GMCSF-R: Granulocyte-macrophage colony-stimulating factor-receptor
GOT: Glutamic oxaloacetic transaminase
GPT: Glutamate pyruvate transaminase
GvHD: Graft versus host disease
HCQ: Hydroxychloroquine
ILD: Interstitial lung disease
ITT: Intention-to-treat
LDH: Lactate dehydrogenase
LRBA: Lipopolysaccharide-responsive and beige-like anchor protein
*MedDRA*: *Medical Dictionary for Regulatory Activities*
NKX2–1: NK2 Homeobox 1
NP B/C: Niemann-Pick disease B/C
OCT: Ocular coherence tomography
SAP: Statistical analysis plan
SFTPB/C: Surfactant protein B/C
TBX4: T-box transcription factor 4
