# Supplementary material for: Prospective evaluation of hydroxychloroquine in pediatric interstitial lung diseases: Study protocol for an investigator-initiated, randomized controlled, parallel-group clinical trial
Source: Trials. 2020 Apr 3;21:307. doi: 10.1186/s13063-020-4188-4 (PMC7118852; doi:10.1186/s13063-020-4188-4)
Supplement: Supplementary file 1 — Additional file 1. a. Time schedule of the HCQ START block and trial assessments. b.Time schedule of the HCQ STOP block and trial assessments. [file 13063_2020_4188_MOESM1_ESM.docx]

| Visit | Screening evaluations and Visit 1 | Visit 2 | Email-/Phone-Visit | Visit 3 | Email-/Phone-Visit | Visit 4 |
| --- | --- | --- | --- | --- | --- | --- |
| Action | „Start of baseline“ | “End of baseline” +  Day 1 of treatment | Every 7 days at home | End of 1st Tx period | Every 7 days at home | “End of trial” |
| Trial day | -28 to -3 | 1 | 7, 14, 21 (+/- 3) | 28 (-4, +7) | 35, 42, 49 (+/- 3) | 56 (-4, +7) |
| Demographics (e.g. sex, age) | X |  |  |  |  |  |
| Patient information, informed consent | X |  |  |  |  |  |
| Previous (last 12 months) and concomitant diseases | X | X | X | X | X | X |
| Previous (last 3 months) and concomitant treatments | X | X | X | X | X | X |
| Inclusion/exclusion criteria | X | X*^5^ |  |  |  |  |
| Pregnancy test ^k^ | X* |  |  | X |  | X |
| Randomization ^a^ | X |  |  |  |  |  |
| Trial medication supply to patient |  | X |  | X |  |  |
| History (cardiotoxicity, hypoglycemia) | X | X |  | X |  | X |
| Physical examination | X | X |  | X |  | X |
| Vital signs (BP, pulse, temp) ^b^ | X | X |  | X |  | X |
| ECG ^j^ | X* |  |  | X |  | X |
| Ophthalmologic review ^j^ | X* |  |  |  |  | X |
| Laboratory ^c^ | X* |  |  | X |  | X |
| pO2, pCO2 (capillary) | X | X*^3^ |  | X*^3^ |  | X*^3^ |
| O2-sat e, in room air | X | X | X | X | X | X |
| Respiratory rate, in room air | X | X | X | X | X | X |
| Retractions | X | X | X | X | X | X |
| Coughing | X | X | X | X | X | X |
| Oxygen demand | X | X | X | X | X | X |
| Chest x-ray | X*^2^ |  |  | X*^4^ |  |  |
| Pulmonary hypertension (Echo) ^j^ | X* |  |  |  |  |  |
| Quality-of-life ^i^ | X* |  |  | X |  | X |
| Health Economics ^h^ | X* |  |  | X |  | X |
| Weight for height ^b^ | X* | X |  | X |  | X |
| Clinical course of lung disease | X*^6^ | X*^6^ | X*^6^ | X*^6^ | X*^6^ | X*^6^ |
| Exacerbation ^o^ | X*^7^ | X*^7^ | X*^7^ | X*^7^ | X*^7^ | X*^7^ |
| Adverse events | X | X | X | X | X | X |
| Drug accountability |  |  |  | X |  | X |
| Additionally in children > 5 years |  |  |  |  |  |  |
| Spirometry / Bodyplethysmography ^d^ | X | X |  | X |  | X |
| 6-minute walking distance (meter) ^g^ | X | X |  | X |  | X |
| O2-saturation before and after 6MWT | X | X |  | X |  | X |
| Borg scale ^f^ | X | X |  | X |  | X |
| Additionally in ventilated patients ^m^ |  |  |  |  |  |  |
| Oxygenation index (OI) ^n^ | X | X | X | X | X | X |
| Duration (h) of mechanical ventilation | X | X | X | X | X | X |
| NO (%), ECLS (VV/VA/ECLA/Flow/time since insertion) | X | X | X | X | X | X |

Additional file 1a: Time schedule of the HCQ START block and trial assessments

Additional file 1b: Time schedule of the HCQ STOP block and trial assessments

|  | Visit | Visit 1  + Screening | Visit 2 | Email-/Phone-Visit | Visit 3 | Email-/ Phone-Visit | Visit 4 | Email-/ Phone-Visit | Visit 5 | Email-/ Phone-Visit | Visit 6 | Email-/Phone-Visit | Visit 7 | Email-/Phone-Visit | Visit 8 |
| --- | --- | --- | --- | --- | --- | --- | --- | --- | --- | --- | --- | --- | --- | --- | --- |
|  | Action | „Begin of baseline“ | “End of baseline” + Day 1 of withdrawal treatment | 2 weeks of withdrawal, at home | 4 weeks of withdrawal treatment | 6 weeks of withdrawal, at home | 8 weeks of withdrawal treatment | 10 weeks of withdrawal, at home | “End of trial” after 12 weeks of withdrawal treatment | 2 weeks of open observation at home | Follow up after 4 weeks of open observation | 6 weeks of withdrawal, at home | Follow up after 8 weeks of open observation | 10 weeks of withdrawal, at home | Follow up after 12 weeks of open observation |
|  | Trial day | -30 to -10 | 1 | 14 (-7, +7) | 28 (-7, +7) | 42 (-7, +7) | 56 (-7, +7) | 70 (-7, +7) | 84 (-7, +7) | 98 (-7, +7) | 112 (-7, +7) | 126 (-7,+7) | 140 (-7, +7) | 154 (-7, +7) | 168 (-7, +7) |
|  | Demographics (e.g. sex, age) | X |  |  |  |  |  |  |  |  |  |  |  |  |  |
|  | Patient information, informed consent | X |  |  |  |  |  |  |  |  |  |  |  |  |  |
|  | Previous (last 12 months) and concomitant diseases | X | X | X | X | X | X | X | X | X | X | X | X | X | X |
|  | Previous (last 3 months) and concomitant treatments | X | X | X | X | X | X | X | X | X | X | X | X | X | X |
|  | Inclusion/exclusion criteria | X | X*^5^ |  |  |  |  |  |  |  |  |  |  |  |  |
|  | Pregnancy test ^K^ | X* |  |  | X |  | X |  | X |  |  |  |  |  |  |
|  | Randomization ^a^ | X |  |  |  |  |  |  |  |  |  |  |  |  |  |
|  | Trial medication supply to patient |  | X |  | X |  | X |  |  |  |  |  |  |  |  |
|  | History (cardiotoxicity, hypoglycemia) | X | X |  | X |  | X |  | X |  | X |  | X |  | X |
|  | Physical examination | X | X |  | X |  | X |  | X |  | X |  | X |  | X |
|  | Vital signs (BP, pulse, temp) ^b^ | X | X |  | X |  | X |  | X |  | X |  | X |  | X |
|  | ECG ^j^ | X* |  |  |  |  |  |  | X |  |  |  |  |  | X |
|  | Ophthalmologic review ^j^ | X* |  |  |  |  |  |  |  |  |  |  |  |  | X *^8^ |
|  | Laboratory ^c^ | X* |  |  | X |  | X |  | X |  | X  (only HCQ drug level) |  | X  (only HCQ drug level) |  | X  (only HCQ drug level) |
|  | pO2, pCO2 (capillary) | X | X*^3^ |  | X*^3^ |  | X*^3^ |  | X*^3^ |  | X*^3^ |  | X*^3^ |  | X*^3^ |
|  | O2-sat e, in room air | X | X | X | X | X | X | X | X | X | X | X | X | X | X |
|  | Respiratory rate, in room air | X | X | X | X | X | X | X | X | X | X | X | X | X | X |
|  | Retractions | X | X | X | X | X | X | X | X | X | X | X | X | X | X |
|  | Coughing | X | X | X | X | X | X | X | X | X | X | X | X | X | X |
|  | Oxygen demand ^L^ | X | X | X | X | X | X | X | X | X | X | X | X | X | X |
|  | Chest x-ray | X*^2^ |  |  |  |  |  |  | X*^4^ |  |  |  |  |  |  |
|  | Pulmonary hypertension (Echo) ^j^ | X* |  |  |  |  |  |  |  |  |  |  |  |  |  |
|  | Quality-of-life ^i^ | X* |  |  | X |  | X |  | X |  | X |  | X |  | X |
|  | Health Economics ^h^ | X* |  |  | X |  | X |  | X |  | X |  | X |  | X |
|  | Weight for height ^b^ | X* | X |  | X |  | X |  | X |  | X |  | X |  | X |
|  | Clinical course of lung disease | X*^6^ | X*^6^ | X*^6^ | X*^6^ | X*^6^ | X*^6^ | X*^6^ | X*^6^ | X*^6^ | X*^6^ | X*^6^ | X*^6^ | X*^6^ | X*^6^ |
|  | Exacerbation ^o^ | X*^7^ | X*^7^ | X*^7^ | X*^7^ | X*^7^ | X*^7^ | X*^7^ | X*^7^ | X*^7^ | X*^7^ | X*^7^ | X*^7^ | X*^7^ | X*^7^ |
|  | Adverse events | X | X | X | X | X | X | X | X | X | X | X | X | X | X |
|  | Drug accountability |  |  |  | X |  | X |  | X |  |  |  |  |  |  |
|  | Additionally in children > 5 years |  |  |  |  |  |  |  |  |  |  |  |  |  |  |
|  | Spirometry / Bodyplethysmography d | X | X |  | X |  | X |  | X |  | X |  | X |  | X |
|  | 6-minute walking distance (meter) ^g^ | X | X |  |  |  |  |  | X |  |  |  |  |  | X |
|  | O2-saturation before and after 6MWT ^g^ | X | X |  |  |  |  |  | X |  |  |  |  |  | X |
|  | Borg scale ^f^ | X | X |  |  |  |  |  | X |  |  |  |  |  | X |
|  | Additionally in ventilated patients m |  |  |  |  |  |  |  |  |  |  |  |  |  |  |
|  | Oxygenation index (OI) ^n^ | X | X | X | X | X | X | X | X | X | X | X | X | X | X |
|  | Duration (h) of mechanical ventilation | X | X | X | X | X | X | X | X | X | X | X | X | X | X |
|  | NO (%), ECLS (VV/VA/ECLA/Flow/time since insertion) | X | X | X | X | X | X | X | X | X | X | X | X | X | X |

X* Tests must be done before first drug dosing

X*^2^ Test can be done according to the opinion of the treating physician

X*^3^ Puncture site should be warm and well perfused ^P^

X*^4^ Chest x-ray is recommended if clinically indicated e.g. to answer the question if the disease is stable

X*^5^ Re-check inclusion criteria 1

X*^6^ (since last visit): Healthy / Sick-better / Sick-same / Sick-worse / Patient died

X*^7^ Pulmonary exacerbation is defined as sustained worsening of the patient’s condition from stable state and beyond normal day to day variations. The exacerbation is rated as indicated below ^o^

X*^8^ This ophthalmologic investigation may be used for patients entering the START block, if START HCQ block is done within 6 months and no HCQ was taken since end of STOP block and the ophthalmological investigation was normal.

^a^ Randomization must occur for logistic reasons as soon as possible after concluding all screening investigations and including the patient into the study. This secures delivery of the study medication in time to any study center.

^b^ Weight (kg) and height (cm) will be measured with shoes and clothes off. Vital signs will be collected after the patient has been at rest for 5 minutes.

^c^ Blood sample should include: Blood count with differential, GOT, GPT, gGT, Creatinine, LDH, Potassium, Creatine kinase, blood glucose.

HCQ drug level (2 ml EDTA blood and 2 ml of Serum blood). Laboratory manual will be provided.

^d^ Spirometry / Bodyplethysmography will be performed pre-bronchodilator in children age ≥ 5 years. (If a child ≤ 5 years is already able to perform the listed investigations (spirometry or bodyplethysmography), these should also be performed and documented at the discretion of the investigator) The following lung function parameters will be assessed: FEV1 % predicted (recorded in L), FEV 1 (L), FVC % predicted (recorded in L), FVC (L); MEF 75 (L/s); MEF 50 (L/s); MEF 25 (L/s); TLC (L); ITGV (L); RV (L); R eff (kPa*s/L); R eff predicted (%). SOP will be provided.

^e^ Measurement of O_2_-Saturation, respiratory rate and O_2_ flow (if necessary) in awake patient at rest:

If not on O_2_: after 5 min at rest, room air, then measure twice over 1 min. each; the measurements have to be at least 1 min. apart; report the stable average value

If on O_2_:

- BEFORE withdrawal (after 5 min at rest with steady state O_2_ supplement, measure over 1 minute)
- Then withdraw O_2_ to obtain steady state O_2_-Sat. in room air (at least 30 seconds without change). Then measure twice over 1 min. each; the measurements have to be at least 1 min. apart; report the stable average value. If the O_2_-Sat falls below SpO_2_<80% place back in oxygen and note as exact value.

Document the time of measurement and state of the patient, usually “awake, at rest”. Note: In babies avoid crying, eating, sleeping during measurement.

Note: try to schedule patients at similar time of the day.

^f^ Borg-Scale: assessment of dyspnoea (0 – 10: 0 = no dyspnoea at all, 5 = severe dyspnoea, 10 = extremely severe dyspnoea)

^g^ Standard exercise testing (6-minute-walk test): SOP will be provided

^h^ Health economics assessment with the use of a special questionnaire which has to be filled out by the parents or adult patients.

^I^ Quality of Life assessment with the use of the questionnaire PedsQL for different age groups which has to be filled out by parents and/or patients

^j^ For Electrocardiography (ECG), ophthalmological review and Echocardiography (Echo) please see Chapter 5. If a routine ophthalmological examination was done within 3 month before Visit 1, no additional ophthalmological exam is required.

^k^ Pregnancy test only has to be performed in girls of childbearing age and only if sexual relations are known or probable. It is at the discretion and responsibility of the attending physician to decide, whether a pregnancy test is necessary or not. The pregnancy test can be performed in the urine or serum.

^L^ The following parameter will also be assessed, if there is oxygen demand:

1. O2 via nasal cannula or via face mask: O2-Flow (l/min)
2. on high-flow nasal cannula: Flow air (l/min), O2-Flow (l/min), FiO2

^m^ The following parameter will also be assessed on ventilation: ventilation method, duration of ventilation (hours/day/only for non-invasive ventilation),

PEEP (cmH_2_O), P_IP_ (cmH_2_O), F_i_O_2,_ T_i_ (sec), M_PAW_ (cmH_2_O) and Oxygenation Index (OI);

In addition, if patients on the heart lung machine (e.g. NO (%), ECLS (VV/VA/ECLA), Flow/time since insertion) will be documented descriptively.

^n^ Calculation of Oxygenation Index (OI): OI = (F_i_O_2_* M_PAW_)/P_a_O2

with M_PAW_ = ((frequency * inspiration time)/60) * ((inspiratory pressure (P_IP_) – PEEP) + PEEP))) ; If PaO2 is not available, PcapO2 can be used.)

^o^ Start date, End date, Patient returned to baseline function? Y/N

1. Increase in respiratory rate? Y/N/not done.

2. Increase or development of dyspnea? Y/N

3. Newly developing or increased abnormalities on chest imaging? Y/N/not done

4. Onset/increase of oxygen demand to attain the individual baseline saturation? Y/N

5. Need for an additional level of ventilatory support (in addition to oxygen)? Y/N

6. Decrease in lung function in children able to perform the tests? Y/N/not done. Give before and after FEV1 (% predicted) _____, FVC (%)______

7. Reduced exercise tolerance (history or in tests)? Y/N

Additional info: 1. Was the patient hospitalized? Y/N, 2. New feeding problem? Y/N, 3. New failure to thrive/weight lost? Y/N, 4. Was there a change in treatment? Y/N

Cause/trigger (multiple selection possible), 1. Infection? Y/N, 2. Exposure to environmental irritant? Y/N, 3. Aspiration? Y/N, 4. Extra-pulmonary processes? Y/N, 5. Changes of treatment prior worsening? Y/N, 6. Poor treatment adherence ? Y/N, 7. Side effect of current medication? Y/N, 8. Psychosocial factors? Y/N. 9. Other? Y/N. Give detail:________

^P^ Capillary blood sample is obtained by the use of a warm, moist towel at a temperature not higher than 42 °C on a puncture site not longer than three to five minutes or use of other vasodilator agent (e.g. Finalgon). Puncture site: Fingertip is recommended (rarely ear lobe or in neonates/infants, heel). If an arterial sample is available, this can substitute the capillary sample.
